# Prospective evaluation of chemotherapy-induced dyslipidemia in early breast cancer: implications for cardiovascular risk

**DOI:** 10.3389/fonc.2025.1677835

**Published:** 2026-01-12

**Authors:** Lenka Rusinova, Jelizaveta Medvedieva, Zuzana Santova, Miriam Kozarova

**Affiliations:** 1 Department of Oncology, Stefan Kukura Hospital, Michalovce, Slovakia; 2 4th Department of Internal Medicine, Faculty of Medicine, Pavol Jozef Šafárik University in Košice, Košice, Slovakia

**Keywords:** breast cancer, cardio-oncology, cardiovascular risk, chemotherapy, dyslipidemia

## Abstract

**Background:**

Cardiovascular disease is a leading cause of long-term mortality among breast cancer survivors, yet chemotherapy-induced dyslipidemia remains an underrecognized component of survivorship care.

**Methods:**

In this prospective, single-center study, 56 women with clinical stage IB–IIIB early breast cancer received standardized anthracycline- and taxane-based chemotherapy. Fasting lipid profiles including low-density lipoprotein cholesterol (LDL-C), high-density lipoprotein cholesterol (HDL-C), total cholesterol, and triglycerides, were assessed at four timepoints: baseline, post–anthracycline–cyclophosphamide phase, end of treatment, and 3-month follow-up. Longitudinal changes were analysed using non-parametric tests and multivariable linear regression.

**Results:**

Baseline dyslipidemia was present in 78.4% of patients. Chemotherapy significantly altered all lipid parameters, with changes in total cholesterol (*p* = 0.004), LDL-C (*p* = 0.007), HDL-C (*p* < 0.001), and triglycerides (*p* = 0.002). The most pronounced alterations occurred during the anthracycline-based phase. Elevated LDL-C and reduced HDL-C levels persisted at follow-up. Lipid changes were not consistently associated with age, BMI, or menopausal status.

**Conclusion:**

Standard chemotherapy induces sustained proatherogenic lipid shifts in women with early breast cancer, independent of baseline cardiovascular risk factors. The unexpectedly high prevalence of dyslipidemia among premenopausal women challenges traditional assumptions of cardiometabolic protection in younger patients. These findings support the integration of lipid monitoring and early cardiovascular risk management into survivorship care.

**Clinical Trial Registration:**

NCT06958783, https://clinicaltrials.gov/study/NCT06958783.

## Introduction

Breast cancer remains the most frequently diagnosed cancer in women, accounting for approximately 2.3 million new cases annually and nearly one-quarter of all female malignancies worldwide (1). Advances in screening and systemic therapies have markedly improved prognosis, with 5-year relative survival rates reaching 99% for stages I–II and 86% for stage III (2). With the growing number of breast cancer survivors, cardiovascular health is gaining importance as a key component of long-term care.

Cardiovascular disease (CVD) is the leading global cause of death, with ischemic heart disease the most significant contributor to premature female mortality worldwide (3). Dyslipidemia, particularly elevated low-density lipoprotein cholesterol (LDL-C) and triglycerides (TG), is a major modifiable risk factor for atherosclerosis and cardiovascular events (4, 5). Among long-term survivors of early-stage breast cancer, the risk of cardiovascular death may exceed the risk of recurrence (6, 7), highlighting the need to integrate cardiometabolic health into routine oncology practice.

Despite progress in targeted therapies and personalized medicine, cytotoxic chemotherapy remains a cornerstone of treatment for early-stage breast cancer, particularly in high-risk subgroups such as node-positive, human epidermal growth factor receptor 2 (HER2)-positive, or triple-negative disease. Beyond established surveillance for anthracycline-induced cardiotoxicity, chemotherapy-induced alterations in lipid metabolism may represent an underrecognized contributor to cardiovascular risk.

A limited number of retrospective studies, mostly conducted in Asian populations, have examined changes in lipid profiles during breast cancer chemotherapy (8–11). These studies have reported heterogeneous findings, emphasizing the need for prospective evaluations using standardized protocols and predefined timepoints.

Emerging experimental evidence suggests that anthracyclines and taxanes, two of the most commonly used chemotherapeutic agents in early breast cancer, may disrupt lipid homeostasis by downregulating genes involved in lipid transport and receptor-mediated clearance (12, 13). However, clinical data supporting these mechanisms remain limited and inconclusive.

To address this gap, we conducted a prospective, single-center study to characterize the metabolic impact of standard anthracycline- and taxane-based regimens and explore their potential implications for cardiovascular risk stratification and survivorship care.

## Methods

### Study participants

Fifty-six adult female patients with newly diagnosed, histologically confirmed invasive breast cancer (clinical stages IB–IIIB) were prospectively enrolled between March 2023 and September 2024 at the Department of Oncology, Stefan Kukura Hospital, Michalovce, Slovakia. Tumor staging was performed according to the 8th edition of the American Joint Committee on Cancer/Union for International Cancer Control (AJCC/UICC) TNM classification (14).

Eligible patients had not received any prior breast cancer treatment and were scheduled to receive either neoadjuvant or adjuvant chemotherapy. Exclusion criteria included the use of lipid-lowering medications at baseline and incomplete administration of the planned chemotherapy regimen.

No patients initiated statins or other lipid-lowering therapies during the study period. To reduce potential behavioral confounders, all patients received standardized nutritional counseling, and smoking cessation was recommended when applicable.

### Treatment protocol

All patients received a standardized chemotherapy backbone consisting of four cycles of doxorubicin (60 mg/m²) and cyclophosphamide (600 mg/m²), administered either every two weeks (dose-dense) or every three weeks (conventional schedule), followed by 12 weekly infusions of paclitaxel (80 mg/m²). The choice between dose-dense and conventional chemotherapy scheduling was based on clinical assessment and aligned with current European Society for Medical Oncology (ESMO) (15) and National Comprehensive Cancer Network (NCCN) (16) guidelines.

Patients with HER2-positive tumors received additional HER2-targeted therapy with trastuzumab, with or without pertuzumab depending on tumor stage (15, 16).

Patients with triple-negative breast cancer (TNBC), defined as hormone receptor–negative and either HER2 negative or HER2-low, additionally received weekly carboplatin at an area under the curve (AUC) of 1.5 (Calvert formula) and pembrolizumab (200 mg every three weeks) (17). Due to the absence of national reimbursement during the study period, only 5 of the 14 patients in this subgroup were treated with pembrolizumab.

### Data collection

Fasting venous blood samples were collected after an overnight fast of 12–14 hours at four predefined timepoints:

− 1–3 days prior to chemotherapy initiation (baseline);
− within 1–3 days after completion of the anthracycline–cyclophosphamide (AC) phase, before the first paclitaxel dose (“after AC”);
− within 1–3 days after the final paclitaxel dose (end of chemotherapy); and
− three months after the last chemotherapy cycle (follow-up).

Serum levels of total cholesterol (TC), TG, LDL-C, and high-density lipoprotein cholesterol (HDL-C) were measured using a validated automated biochemical analyzer in the hospital laboratory. Lipid abnormalities were defined according to the 2021 European Society of Cardiology/European Atherosclerosis Society (ESC/EAS) Guidelines (18): TC ≥ 5.0 mmol/L, TG ≥ 1.7 mmol/L, LDL-C ≥ 3.0 mmol/L, and HDL-C < 1.2 mmol/L in women.

Baseline clinical and pathological characteristics were collected, including age, body mass index (BMI), menopausal status, tumor (T) and nodal (N) stage, molecular subtype, and cardiovascular risk factors (**Supplementary Table S1**). Cardiovascular risk factors were defined as the presence of hypertension, smoking, diabetes mellitus, known dyslipidemia, or a family history of premature CVD—defined as myocardial infarction or stroke in a first-degree male relative before age 55 or a first-degree female relative before age 65, in accordance with ESC/EAS criteria.

### Ethics statement

The study was approved by the institutional Ethics Committee (LRS/28/02/2023) and conducted in accordance with the Declaration of Helsinki. Written informed consent was obtained from all participants prior to enrollment.

The study is registered at ClinicalTrials.gov (identifier: NCT06958783).

### Statistical analysis

All statistical analyses were performed using IBM SPSS Statistics for Windows, version 29.0 (IBM Corp., Armonk, NY, USA). Continuous variables were expressed as medians with interquartile ranges (IQR) or means ± standard deviations (SD), as appropriate. Categorical variables were summarized as absolute frequencies and percentages. The Shapiro–Wilk test was used to assess normality of distribution for continuous variables.

Given the small sample size and the non-normal distribution of several lipid parameters—particularly TG and HDL-C—non-parametric methods were applied for most analyses. Repeated measurements of lipid levels (TC, LDL-C, HDL-C, TG) across four predefined timepoints were analyzed using the Friedman test. When global significance was detected, pairwise *post hoc* comparisons were conducted using the Wilcoxon signed-rank test with Bonferroni correction. As three pairwise comparisons were performed per lipid parameter, the corrected significance threshold was set at *p* < 0.017.

Associations between lipid changes and clinical variables were examined through subgroup comparisons and correlation analyses. Between-group comparisons (according to menopausal status, BMI category, age group, smoking status, and cardiovascular risk factor burden) were carried out using the Mann–Whitney U test for two-group comparisons, or the Kruskal–Wallis test for three or more groups. Correlations with continuous variables (age, BMI, number of cardiovascular risk factors) were assessed using Spearman’s rank correlation coefficients.

To identify independent predictors of lipid changes, multiple linear regression models were constructed. The dependent variables were changes in LDL-C and HDL-C from baseline. Independent variables included age, menopausal status, BMI, smoking status, number of cardiovascular risk factors, and selected chemotherapy agents (trastuzumab, pertuzumab, carboplatin, pembrolizumab). TC and TG were excluded from the multivariable models due to the absence of statistically significant associations in prior analyses.All statistical tests were two-sided, and *p*-values < 0.05 were considered statistically significant.

## Results

A total of 56 women with newly diagnosed early-stage breast cancer were prospectively enrolled. Five were excluded due to incomplete chemotherapy or loss to follow-up, resulting in a final cohort of 51 patients. In addition to standard chemotherapy, 21 patients (41.2%) received HER2-targeted therapy with trastuzumab, with or without pertuzumab, and 5 (9.8%) received carboplatin combined with pembrolizumab.

The median age was 54 years (range, 39–80); 49.0% of patients were pre- or perimenopausal, and 51.0% were postmenopausal. Baseline characteristics are presented in **Supplementary Table S1**. At diagnosis, 78.4% of patients had at least one lipid abnormality, most commonly elevated total cholesterol (62.7%) and LDL-C (60.8%), followed by hypertriglyceridemia (41.2%) and reduced HDL-C (19.6%).

**Figure 1:**
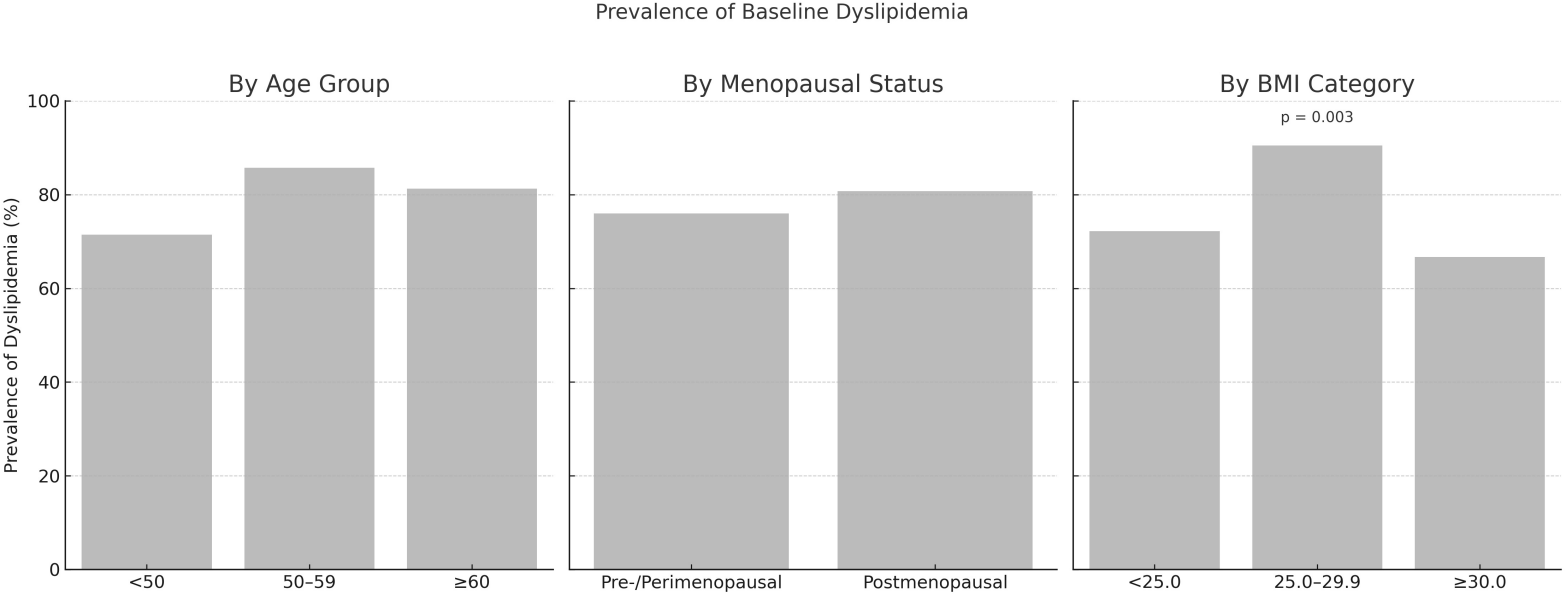
Prevalence of baseline dyslipidemia by age group, menopausal status, and body mass index (BMI) category.

The prevalence of dyslipidemia did not differ significantly by menopausal status (76.0% in pre-/perimenopausal vs. 80.8% in postmenopausal; *p* = 1.000) or across age categories (<50, 50–59, ≥60 years; *p* = 0.355). However, a significant difference was observed across BMI categories (<25.0, 25.0–29.9, ≥30.0 kg/m²; *p* = 0.003), with the highest prevalence among overweight individuals (**Figure 1**).

**Figure 2:**
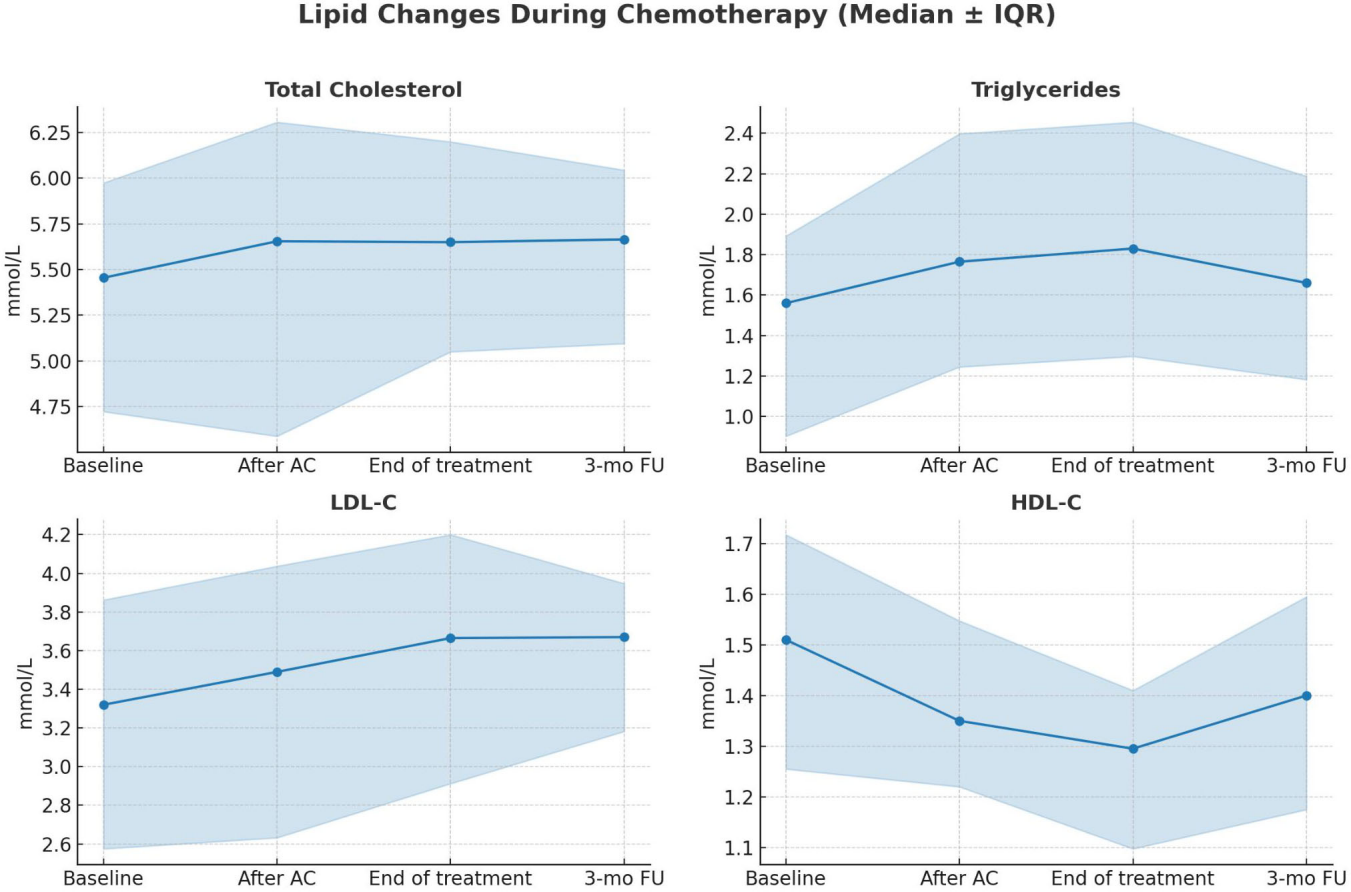
Changes in lipid parameters during chemotherapy. Median values with interquartile ranges are shown for total cholesterol, triglycerides, LDL-cholesterol, and HDL-cholesterol at baseline, after AC chemotherapy, at end of treatment, and at 3-month follow-up. AC, anthracycline–cyclophosphamide; LDL-C, low-density lipoprotein cholesterol; HDL-C, high-density lipoprotein cholesterol.

Lipid levels changed significantly over the course of chemotherapy, with distinct temporal trends (**Figure 2**, **Supplementary Table S2**). Overall changes were observed in TC (*p* = 0.004), LDL-C (*p* = 0.007), HDL-C (*p* < 0.001), and TG (*p* = 0.002). TC increased modestly, peaking at the end of chemotherapy and slightly declining at follow-up. LDL-C rose significantly from baseline to the end of treatment (*p* = 0.015), while HDL-C decreased progressively (baseline to post-AC, *p* = 0.004; end of chemotherapy, *p* < 0.001; follow-up, *p* = 0.013). Although TG levels showed significant overall variation, pairwise comparisons did not remain significant after correction for multiple testing.

To further elucidate the timing of these changes, lipid levels were compared between the AC and paclitaxel phases. The most pronounced alterations occurred during the AC phase, including increases in LDL-C (*p* = 0.048) and triglycerides (*p* = 0.024), and a decrease in HDL-C (*p* = 0.001). No significant changes were observed during the paclitaxel phase (all *p* > 0.05), suggesting that lipid disturbances were primarily induced by early chemotherapy. Lipid trajectories are detailed in **Supplementary Table S2**; **Supplementary Tables S1**, **S2**.

To assess the clinical impact of lipid changes, we estimated the proportion of patients who would potentially qualify for lipid-lowering therapy based on LDL-C thresholds. Following ESC/EAS recommendations (18), we used LDL-C ≥3.0 mmol/L as a pragmatic proxy for potential indication of lipid-lowering therapy. At baseline, 31 of 51 patients (60.8%) met this criterion, which increased to 37 (72.5%) at the end of treatment and 39 (76.5%) at 3-month follow-up. When applying a broader definition of dyslipidemia (≥1 abnormal lipid parameter: LDL-C ≥3.0 mmol/L, total cholesterol ≥5.0 mmol/L, triglycerides ≥1.7 mmol/L, or HDL-C <1.2 mmol/L), the proportions were 40 (78.4%), 48 (94.1%), and 47 (92.2%), respectively. Overall, 13 patients (25%) developed new or additional lipid abnormalities during the study period.

Changes in lipid levels were not significantly associated with baseline characteristics such as age, BMI, menopausal status, or smoking. However, an inverse correlation between TG elevation and the number of cardiovascular risk factors was observed (*ρ* = –0.298, *p* = 0.033). A borderline association between HDL-C decline and BMI was also noted (*ρ* = 0.254, *p* = 0.072). No other relevant associations were identified (**Supplementary Table S3**).

Multivariable linear regression models were constructed for LDL-C and HDL-C, which exhibited the most consistent temporal changes in univariate analyses (**Supplementary Table S3**). LDL-C increases were significantly associated with the use of anti-HER2 therapy (*p* = 0.021) and pembrolizumab-containing regimens (*p* = 0.002). These associations remained significant after adjustment, although interpretation is limited by small sample sizes in these subgroups. No independent predictors of HDL-C change were identified, although borderline trends were observed for BMI (*p* = 0.087) and immunotherapy exposure (*p* = 0.081). No consistent predictive associations were found for baseline clinical variables.

## Discussion

This prospective study demonstrated significant and dynamic alterations in lipid metabolism during chemotherapy. All major lipid parameters (TC, LDL-C, HDL-C, and TG) underwent overall changes throughout the treatment course, with distinct temporal patterns observed across different phases of chemotherapy.

The most pronounced changes occurred during the AC phase, characterized by an increase in LDL-C and TG and a reduction in HDL-C, while no further significant changes were observed during the paclitaxel phase. Importantly, these alterations, particularly elevated LDL-C and reduced HDL-C, persisted three months after treatment completion, suggesting a sustained treatment-related metabolic effect.

Alterations of lipid metabolism are increasingly recognized as an important aspect of cancer biology, arising from tumor-induced oxidative stress, chronic inflammation, and systemic metabolic reprogramming (19, 20). In breast cancer specifically, accumulating evidence highlights lipid metabolic reprogramming as a key feature supporting tumor growth, progression, and adaptation to oxidative stress. Breast cancer cells enhance lipid uptake and synthesis, remodel lipoprotein metabolism, and exploit lipid-derived energy to sustain proliferation and invasion (21, 22). Clarifying the contribution of tumor-driven metabolic alterations to baseline dyslipidemia in breast cancer patients represents an important direction for future research.

The existing evidence on chemotherapy-induced lipid changes largely stems from retrospective studies, with findings that remain inconsistent in the current literature. Tian et al. observed significant elevations in LDL-C and TG, along with reduced HDL-C in younger women, with partial normalization six months post-treatment (8). He et al. reported increases in LDL-C and TC, but no significant changes in HDL-C or TG (9). Xu et al. documented persistent dyslipidemia for up to one year, particularly in patients treated with anthracycline-based regimens (10). These inconsistencies likely reflect methodological variation, including population heterogeneity, differences in chemotherapy protocols, and variable timing of lipid assessments.

By applying standardized lipid assessments at predefined timepoints and using a uniform anthracycline- and taxane-based regimen, the present study offers improved temporal resolution and provides new insight into the trajectory of lipid changes during and after chemotherapy.

No significant associations were identified between chemotherapy-induced lipid changes and baseline clinical characteristics such as age, BMI, menopausal status, or smoking status. A modest inverse correlation was observed between TG elevation and the number of cardiovascular risk factors, and a borderline positive association emerged between BMI and HDL-C decline. The TG correlation may reflect a ceiling effect, previously described in cardiometabolic literature, where patients with multiple risk factors already have elevated TG levels at baseline, leaving less potential for further increases during treatment (23). Chronic low-grade inflammation and insulin resistance, frequently observed in individuals with multiple cardiometabolic risk factors, may also contribute to persistently elevated TG and a reduced metabolic response to chemotherapy (24).

In multivariable analysis, baseline clinical characteristics showed limited predictive value for HDL-C dynamics, although borderline trends were noted for BMI and exposure to immunotherapy. In contrast, LDL-C elevation was significantly associated with the use of anti-HER2 therapy (*p* = 0.021) and pembrolizumab-containing regimens (*p* = 0.002), suggesting that these agents may contribute to lipid dysregulation via distinct pharmacologic mechanisms. This interpretation is supported by preclinical data implicating HER2-dependent signaling in trastuzumab-associated lipid effects (25), and by clinical reports linking pembrolizumab-induced hypothyroidism to impaired lipid clearance and subsequent elevations in LDL-C and TG (26, 27).

Cytotoxic agents such as doxorubicin and paclitaxel also exert direct effects on lipid metabolism. Doxorubicin downregulates HDL synthesis by suppressing ATP-binding cassette transporter A1 (ABCA1) via inhibition of liver X receptor alpha (LXRα) and peroxisome proliferator-activated receptor alpha (PPARα) (12). Paclitaxel impairs LDL-C clearance by reducing LDL receptor expression (13). These mechanistic insights support the interpretation of chemotherapy-induced dyslipidemia as a pharmacologically mediated phenomenon, largely independent of preexisting clinical factors.

In addition to pharmacologic mechanisms, behavioral factors have also been explored. However, recent evidence indicates that dietary modifications during chemotherapy may have limited impact on lipid metabolism. A study by Pedersini et al. showed that although breast cancer patients reported changes in food preferences and eating habits during treatment, these adjustments did not translate into clinically meaningful improvements in lipid profiles (28). The persistence of dyslipidemia in this context reinforces the interpretation of a predominantly chemotherapy-driven metabolic effect.

Given the limited impact of behavioral factors, evolving perspectives in lipid biology offer additional insight into the patterns observed. HDL-C, once considered directly cardioprotective, is now viewed primarily as a marker of cardiometabolic health (29, 30). The inverse relationship between TG and HDL-C reflects an inflammatory state, wherein elevated TG may serve as an indicator of subclinical inflammation (29). Accordingly, recent prevention guidelines emphasize TG reduction and non-HDL-C monitoring as therapeutic targets (31).

These pathophysiological insights are underscored by the notable prevalence of baseline dyslipidemia observed among premenopausal women in our cohort, challenging the traditional view of metabolic protection in younger populations. Contrary to expectations, baseline dyslipidemia did not differ significantly between pre-/perimenopausal and postmenopausal women (76.0% vs. 80.8%, p=1.000), despite the well-established adverse lipid changes typically associated with the menopausal transition (32, 33). This pattern may be partly explained by the rising incidence of obesity (34) and polycystic ovary syndrome (PCOS) (35), both of which are associated with insulin resistance and a characteristic dyslipidemic phenotype—elevated TG, small dense LDL particles, and reduced HDL-C (36).

Among premenopausal women in our study, LDL-C and TG levels were already elevated at diagnosis and further increased during the AC phase, amplifying the atherogenic burden early in the treatment course. Beyond this subgroup, in the overall cohort more than 60% of patients already met ESC/EAS criteria for potential lipid-lowering therapy at baseline, and this proportion increased further during chemotherapy. Overall, one quarter of patients developed new or additional lipid abnormalities, underscoring that treatment-induced dyslipidemia may have immediate clinical implications for cardiovascular prevention.

This perspective aligns with recent recommendations from the European Atherosclerosis Society, which emphasize sex-specific cardiovascular prevention strategies and individualized lipid targets for women (37). Our findings suggest that breast cancer survivors, particularly those with persistent dyslipidemia and multiple cardiovascular risk factors, represent a high-priority group for early cardiometabolic intervention. Integrating lipid surveillance and risk-adapted prevention strategies into standard oncology care may offer a pragmatic approach to improving long-term cardiovascular outcomes in this growing patient population.

### Limitations

As a single-center pilot study with a modest sample size, our findings should be interpreted cautiously. Stratification of lipid alterations according to therapy outcome could help disentangle cancer-related from treatment-induced effects, but was not feasible due to the limited sample size and short follow-up. Nevertheless, the prospective design, uniform treatment protocol, and standardized timepoints strengthen internal validity. Residual confounding, particularly from unmeasured behavioral factors, cannot be entirely excluded. Patients identified with persistent dyslipidemia were referred to primary care or internal medicine specialists for ongoing cardiovascular management and remain under structured long-term follow-up.

### Clinical implications

− Standard chemotherapy for early-stage breast cancer induces sustained proatherogenic shifts in lipid profiles, most notably during the anthracycline–cyclophosphamide phase.
− These alterations, particularly persistent elevations in LDL-C and TG together with reductions in HDL-C, may increase long-term cardiovascular risk.
− The lipid changes appear largely independent of age, body mass index, or menopausal status, indicating a systemic, treatment-driven mechanism.
− The unexpectedly high prevalence of dyslipidemia among premenopausal women challenges assumptions of cardiometabolic protection in younger patients.
− Findings support integrating lipid monitoring and cardiovascular risk assessment into routine care, starting at diagnosis and extending through treatment.
− Early implementation of preventive strategies, including consideration of lipid-lowering therapy, may help mitigate long-term cardiovascular morbidity in breast cancer survivors.

## Conclusion

Chemotherapy-induced dyslipidemia is a clinically relevant yet underrecognized consequence of curative treatment for early-stage breast cancer. In this prospective study, most patients presented with baseline lipid abnormalities, and chemotherapy further exacerbated the atherogenic profile, most notably through increases in LDL-C and reductions in HDL-C, regardless of age, BMI, or menopausal status.

These findings highlight the need to incorporate structured lipid monitoring into standard breast cancer care. Early identification of women at increased cardiovascular risk could enable timely, risk-adapted interventions. As the population of breast cancer survivors continues to grow, integrating cardiometabolic surveillance into routine follow-up may help improve long-term outcomes. Future trials should evaluate whether early lipid-lowering strategies can effectively reduce cardiovascular morbidity in this setting.

## Data Availability

The original contributions presented in the study are included in the article/**Supplementary Material**. Further inquiries can be directed to the corresponding author.
