# Supplementary material for: Prospective evaluation of chemotherapy-induced dyslipidemia in early breast cancer: implications for cardiovascular risk
Source: Front Oncol. 2026 Jan 12;15:1677835. doi: 10.3389/fonc.2025.1677835 (PMC12832420; doi:10.3389/fonc.2025.1677835)
Supplement: Supplementary file 1 [file Table1.docx]

# Supplementary Table S1. Changes in serum lipid parameters during and after chemotherapy (Mean ± SD)

| Lipid parameter | Baseline | After AC | End of CT | 3 months post CT |
| --- | --- | --- | --- | --- |
| total cholesterol (mmol/L) | 5.35 ± 0.98 | 5.56 ± 1.16 | 5.67 ± 1.03 | 5.56 ± 0.93 |
| LDL cholesterol (mmol/L) | 3.28 ± 0.93 | 3.43 ± 0.97 | 3.59 ± 0.94 | 3.50 ± 0.80 |
| HDL cholesterol (mmol/L) | 1.51 ± 0.45 | 1.35 ± 0.33 | 1.29 ± 0.29 | 1.39 ± 0.36 |
| triglycerides (mmol/L) | 1.94 ± 1.65 | 2.12 ± 1.45 | 2.07 ± 1.08 | 1.87 ± 1.06 |

Values are presented as mean ± standard deviation. Timepoints: baseline, after AC, end of CT, and 3-month follow-up.

Abbreviations: SD, standard deviation; LDL, low-density lipoprotein; HDL, high-density lipoprotein; AC, anthracycline–cyclophosphamide; CT, chemotherapy.
