# Supplementary material for: Prospective evaluation of chemotherapy-induced dyslipidemia in early breast cancer: implications for cardiovascular risk
Source: Front Oncol. 2026 Jan 12;15:1677835. doi: 10.3389/fonc.2025.1677835 (PMC12832420; doi:10.3389/fonc.2025.1677835)
Supplement: Supplementary file 2 [file Table2.docx]

# Supplementary Table S2: Post-hoc Wilcoxon signed-rank test results for serum lipid changes

| Lipid parameter | T1 vs T2 | T1 vs T3 | T1 vs T4 |
| --- | --- | --- | --- |
| Total cholesterol (mmol/L) | 0.327 | 0.068 | 0.232 |
| LDL cholesterol (mmol/L) | 0.229 | 0.015 | 0.067 |
| HDL cholesterol (mmol/L) | 0.004 | <0.001 | 0.013 |
| Triglycerides (mmol/L) | 0.327 | 0.136 | 0.670 |

Timepoints: T1 = baseline; T2 = after AC; T3 = end of CT; T4 = 3 months post CT. *p*-values represent comparisons of each timepoint with baseline (T1) using the Wilcoxon signed-rank test. Bonferroni correction was applied for multiple comparisons. *p*-values < .05 were considered statistically significant.

Abbreviations: LDL-C, low-density lipoprotein cholesterol; HDL-C, high-density lipoprotein cholesterol; TG, triglycerides; AC, anthracycline–cyclophosphamide; CT, chemotherapy.
