# Supplementary material for: Prospective evaluation of chemotherapy-induced dyslipidemia in early breast cancer: implications for cardiovascular risk
Source: Front Oncol. 2026 Jan 12;15:1677835. doi: 10.3389/fonc.2025.1677835 (PMC12832420; doi:10.3389/fonc.2025.1677835)
Supplement: Supplementary file 3 [file Table3.docx]

# Supplementary Table S3. Spearman correlation analysis between baseline clinical variables and chemotherapy-induced lipid changes

| Clinical Variable | Lipid Change | Spearman’s ρ | *p*-value |
| --- | --- | --- | --- |
| Age | Δ TC | -0.023 | 0.875 |
| Age | Δ TG | 0.069 | 0.631 |
| Age | Δ LDL-C | -0.024 | 0.869 |
| Age | Δ HDL-C | 0.079 | 0.582 |
| BMI | Δ TC | -0.119 | 0.405 |
| BMI | Δ TG | -0.143 | 0.317 |
| BMI | Δ LDL-C | -0.07 | 0.624 |
| BMI | Δ HDL-C | 0.254 | 0.072 |
| Number of cardiovascular risk factors | Δ TC | -0.008 | 0.955 |
| Number of cardiovascular risk factors | Δ TG | -0.298 | 0.033 |
| Number of cardiovascular risk factors | Δ LDL-C | 0.064 | 0.656 |
| Number of cardiovascular risk factors | Δ HDL-C | 0.172 | 0.227 |
| Smoking status | Δ TC | -0.049 | 0.733 |
| Smoking status | Δ TG | -0.334 | 0.017 |
| Smoking status | Δ LDL-C | 0.047 | 0.742 |
| Smoking status | Δ HDL-C | 0.255 | 0.071 |
| Menopausal status | Δ TC | -0.024 | 0.867 |
| Menopausal status | Δ TG | 0.199 | 0.163 |
| Menopausal status | Δ LDL-C | -0.124 | 0.386 |
| Menopausal status | Δ HDL-C | 0.112 | 0.434 |

Spearman correlation coefficients (ρ) between clinical variables and changes in lipid parameters. No consistent significant associations were observed.

Abbreviations: LDL-C, low-density lipoprotein cholesterol; HDL-C, high-density lipoprotein cholesterol; TG, triglycerides; TC, total cholesterol; BMI, body mass index.
