# Supplementary material for: Prospective evaluation of chemotherapy-induced dyslipidemia in early breast cancer: implications for cardiovascular risk
Source: Front Oncol. 2026 Jan 12;15:1677835. doi: 10.3389/fonc.2025.1677835 (PMC12832420; doi:10.3389/fonc.2025.1677835)
Supplement: Supplementary file 4 [file Table4.docx]

# Supplementary Table S4. Multivariable Linear Regression Models for Predictors of Chemotherapy-Induced Changes in LDL-C and HDL-C

# A.Change in LDL-C (ΔLDL)

| Covariate | Coefficient (B) | 95% CI | *p*-value |
| --- | --- | --- | --- |
| Postmenopausal (vs pre-/perimenopausal) | -0.42 | [-0.99, 0.15] | 0.141 |
| Smoker (vs non-smoker) | -0.17 | [-0.76, 0.42] | 0.568 |
| CHT + antiHER2 treatment | 0.49 | [0.08, 0.90] | 0.021 |
| CHT + carboplatin | 0.51 | [-0.02, 1.04] | 0.061 |
| CHT + carboplatin +  pembrolizumab | 1.06 | [0.41, 1.71] | 0.002 |
| Age | 0.01 | [-0.02, 0.04] | 0.493 |
| Body Mass Index | 0.01 | [-0.03, 0.05] | 0.653 |
| Cardiovascular risk factor count | 0.00 | [-0.25, 0.25] | 0.987 |

## B. Change in HDL-C (ΔHDL)

| Covariate | Coefficient (B) | 95% CI | *p-*value |
| --- | --- | --- | --- |
| Postmenopausal (vs pre-/perimenopausal) | -0.01 | [-0.30, 0.29] | 0.965 |
| Smoker (vs non-smoker) | 0.22 | [-0.09, 0.52] | 0.154 |
| CHT + antiHER2 treatment | 0.11 | [-0.10, 0.32] | 0.299 |
| CHT + carboplatin | 0.03 | [-0.25, 0.31] | 0.826 |
| CHT + carboplatin +  pembrolizumab | 0.30 | [-0.04, 0.63] | 0.081 |
| Age | 0.00 | [-0.01, 0.02] | 0.916 |
| Body Mass Index | 0.02 | [-0.00, 0.04] | 0.087 |
| Cardiovascular risk factor count | -0.04 | [-0.17, 0.09] | 0.502 |

Multivariable linear regression models were conducted to evaluate predictors of changes in LDL-C and HDL-C between baseline and 3 months post-chemotherapy. Covariates included age, BMI, menopausal status, smoking status, number of cardiovascular risk factors, and treatment group (CHT, CHT + antiHER2 treatment, CHT + carboplatin, and CHT + carboplatin + pembrolizumab). The standard chemotherapy group served as the reference.

Abbreviations: LDL-C, low-density lipoprotein cholesterol; HDL-C, high-density lipoprotein cholesterol; BMI, body mass index; CI, confidence interval.
